# Low Frequency of Poultry-to-Human H5N1 Transmission, Southern Cambodia, 2005

**DOI:** 10.3201/eid1210.060424

**Published:** 2006-10

**Authors:** Sirenda Vong, Benjamin Coghlan, Sek Mardy, Davun Holl, Heng Seng, Sovann Ly, Megge J. Miller, Philippe Buchy, Yves Froehlich, Jean Baptiste Dufourcq, Timothy M. Uyeki, Wilina Lim, Touch Sok

**Affiliations:** * Institut Pasteur in Cambodia, Phnom Penh, Cambodia;; † World Health Organization, Phnom Penh, Cambodia;; ‡ Burnet Institute, Melbourne, Victoria, Australia;; § Australian National University, Canberra, Australian Capital Territory, Australia;; ¶ Ministry of Agriculture, Forestry and Fisheries, Phnom Penh, Cambodia;; # Ministry of Health, Phnom Penh, Cambodia;; ** Food and Agricultural Organization of the United Nations, Phnom Penh, Cambodia;; †† Calmette Hospital, Phnom Penh, Cambodia;; ‡‡ Centers for Disease Control and Prevention, Atlanta, Georgia, USA;; §§ Hong Kong Department of Health, Hong Kong Special Administrative Region, People's Republic of China

**Keywords:** H5N1, Cambodia, microneutralization, seroprevalence, South East Asia, poultry mortality, risk factors, South East Asia, research

## Abstract

To understand transmission of avian influenza A (H5N1) virus, we conducted a retrospective survey of poultry deaths and a seroepidemiologic investigation in a Cambodian village where a 28-year-old man was infected with H5N1 virus in March 2005. Poultry surveys were conducted within a 1-km radius of the patient's household. Forty-two household flocks were considered likely to have been infected from January through March 2005 because >60% of the flock died, case-fatality ratio was 100%, and both young and mature birds died within 1 to 2 days. Two sick chickens from a property adjacent to the patient's house tested positive for H5N1 on reverse transcription–PCR. Villagers were asked about poultry exposures in the past year and tested for H5N1 antibodies. Despite frequent, direct contact with poultry suspected of having H5N1 virus infection, none of 351 participants from 93 households had neutralizing antibodies to H5N1. H5N1 virus transmission from poultry to humans remains low in this setting.

## Abstract 2

Transmission is low despite extensive human contact with poultry.

From its identification in poultry in the People's Republic of China in 1996 and outbreak among commercial farms and live poultry markets in Hong Kong in 1997 (*1*), highly pathogenic avian influenza A (H5N1) virus has become an unprecedented epizootic and spread to domestic poultry and wild bird populations in Asia (*2**,**3*), the Middle East, Europe, and Africa (*4*). This epizootic has affected farmers and the agricultural industry, claimed human lives, and raised the specter of a global influenza pandemic, perhaps even beyond the scale of the devastating 1918 "Spanish" influenza pandemic (*5*).

In Cambodia, highly pathogenic H5N1 was first reported in poultry in January 2004 (*6*). Of 92 poultry outbreaks that year, 15 were confirmed by isolation of H5N1 viruses (*7*). During the first 4 months of 2005, 4 fatal human H5N1 cases were detected in Kampot Province, southeast Cambodia (*8*). These human cases occurred contemporaneously with unreported outbreaks of high deaths among chicken flocks throughout Kampot Province. However, H5N1 virus was confirmed in both a person and poultry in only 1 area of Kampot Province, a village in Banteay Meas District, ≈20 km from the Vietnam border and 15 km from the household of the first confirmed human H5N1 case-patient in Cambodia.

The patient from Banteay Meas District was a 28-year-old male farmer in whom a low-grade fever and dizziness developed on March 17, 2005. Approximately 1 week before he became sick, chickens at his home suddenly began dying. His family reported that he plucked at least 1 chicken and ate poultry that had died of illness suggestive of H5N1 disease. He may also have collected dead birds. On the third day of his illness, nonproductive cough, shortness of breath, and watery diarrhea developed. Two days later, he was transported to a Phnom Penh hospital. His condition rapidly deteriorated, and he died the next day despite mechanical ventilation and inotropic support. H5N1 virus infection was confirmed by reverse transcriptase (RT)–PCR from blood; tracheal aspirates; and nasopharynx, throat, and rectal swab specimens collected during his hospitalization (Institut Pasteur – Cambodia, unpub. data).

The farmer's rural village provided a setting in which we could study the epidemiologic features of H5N1 virus in poultry and humans. We report results of a retrospective study of poultry deaths and an H5N1 antibody seroepidemiologic investigation among residents of this village in Banteay Meas District, Kampot Province, Cambodia.

## Methods

### Retrospective Poultry Death Survey

We conducted an immediate investigation in response to notification of the confirmed human H5N1 case in Banteay Meas District. From March 25 through 27, 2005, all households located within a radius of 1 km from the H5N1 case-patient's household were mapped and positioned with a hand-held global positioning system (Garmin, Olathe, KS, USA). We collected information on illness suggestive of H5N1 among animals in each household by interviewing the head of the family with a standardized questionnaire. Households where the head of the family was not at home or could not be found were omitted.

A household chicken flock was considered likely to have been infected by H5N1 virus during the previous 6 months if all of the following characteristics were reported: flock death >60%, 100% case-fatality ratio, and sudden death of young and mature birds within 1 or 2 days of becoming sick. We collected sick poultry and carcasses for H5N1 virus testing. Cloacal swabs of 10 to 14 randomly selected, live, healthy poultry were also collected from each household where birds remained.

### Seroepidemiologic Investigation

We conducted a seroepidemiologic investigation June 3–7, 2005, ≈2 months after the village reported high poultry deaths. It consisted of interviews of household members with a standardized 39-question questionnaire on demographic information and data on specific exposures to animals and the environment during the last 12 months; a 5-mL venous blood specimen was also collected from participants. Four investigation teams of 3 members each visited all households in 4 different directions, starting from the household of the confirmed human case-patient, until 300 participants were enrolled in the study. Each household was visited once, and no further attempts were made to interview absent adult household members. The sample size was estimated to have a 95% chance of detecting >1 seropositive person, if one assumes a 2% prevalence of H5N1 antibodies in the village. Written informed consent was obtained from adults or from a parent or guardian for children <18 years of age. The study was approved by the Cambodian Ethics Committee.

### Laboratory Methods

All animal samples were placed into viral transport medium in sterile tubes in the field, kept cold, and transported daily to the National Animal Health Laboratory of the Ministry of Agriculture, Forestry and Fisheries in Phnom Penh. Cloacal specimens and organ samples from sick and dying poultry were tested for influenza A with an indirect fluorescent antibody assay. Positive results were forwarded to the virology unit of the Institut Pasteur in Cambodia for confirmation with real-time RT-PCR to detect H5 viral RNA. Human blood specimens were centrifuged, and sera were aliquoted and frozen at –80°C. Sera were shipped on dry ice to the Hong Kong Government Virus Unit Laboratory for detection of H5N1 neutralizing antibodies by microneutralization assay and confirmatory Western blot assay. Serologic evidence of H5N1 virus infection was defined as an H5N1 neutralizing antibody titer >80 with a confirmatory Western blot assay (*9*).

### Statistical Analyses

The position codes of all surveyed households were entered into ArcGIS version 9.0 (ESRI Systems, Redlands, CA, USA). We used the space-time scan statistic to determine the cluster of households most likely to have been affected by H5N1 virus in the previous 6 months. Analysis was performed with SaTScan version 5.1.3 (*10*); cases were assumed to follow a Poisson distribution. The space-time statistic is defined by a cylindrical window with a circular geographic base and height pertaining to time. The window is moved in space and time for each geographic location and size. We obtained several overlapping cylinders of different sizes and shapes covering the study area; each cylindrical window reflected a possible cluster. The most likely cluster is the one least likely to have occurred by chance, according to the maximum likelihood ratio test statistic. Individual and household data were entered into EpiData version 3.02 (EpiData Association, Odense, Denmark) and validated with a duplicate data file. STATA version 8.0 (StataCorp LP, College Station, TX, USA) was used for all statistical analyses. Odds ratios were estimated with bivariate logistic regression. Independent associations between demographic and behavioral data and households that were likely to be affected by H5N1 in poultry were also analyzed by logistic regression models. We accounted for the cluster effect of households with STATA's "cluster" option for logistic regression, which specifies that observations are independent across households but not within households. For multivariate analysis, variables with a p value <0.1 were retained in the models. Selected variables whose correlation coefficient was >0.4, which indicates colinearity between these variables, were not included in the logistic regression model.

These investigations were conducted as a collaborative effort between the Cambodian Ministry of Health and Ministry of Agriculture, Forestry and Fisheries, Institut Pasteur in Cambodia, the World Health Organization, Hong Kong Public Health Department, the Centers for Disease Control and Prevention, and the Food and Agricultural Organization of the United Nations.

## Results

### Poultry Deaths in the Village

Of 194 households located within 1 km of the H5N1 patient's house, 163 (84%) had occupants who were home at the time of the survey. No household refused to participate.

**Figure 1:**
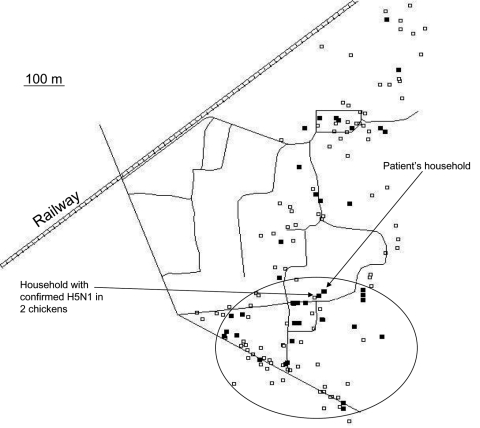
Clustering of 25 households with a high likelihood of avian influenza H5N1 (35%) in chickens, February 27–March 26, 2005, southern Cambodia. White squares indicate visited households without chicken deaths, and black squares indicate households with a chicken flock that was probably infected with H5N1 virus. The cluster is indicated by the circle.

**Figure 2:**
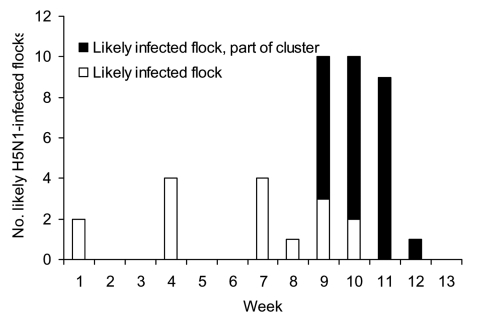
Infected flocks detected by week of reporting period, January 1–March 26, 2005, southern Cambodia. Cluster refers to households within the circle on Figure 1.

Among interviewed households, 155 (95%) raised chickens (median 20, range 1–80 per household) and 52 (32%) raised ducks (median 4, range 1–50). Fifty households (31%) reared both ducks and chickens. Sixty-three households owned pigs (range 1–4 animals). From January 1 through March 26, 2005, 102 (66%) of 155 households reported deaths of chickens. Of these households, 73 (72%) recorded deaths during the last 4 weeks of this 3-month period (Figure 1 and Figure 2). The median poultry flock death ratio was 90% (range 4%–100%). According to our definition, 42 households were likely to have had an outbreak of H5N1, for an overall attack rate of 27% among households with chickens. Flock death ratio was >80% in 31 of these households. The initial mean flock size in households likely to have had H5N1 in chickens was significantly larger than in households without chicken deaths (31 vs. 20, p = 0.003).

Eleven households with a high likelihood of H5N1 (35%) in chickens also owned ducks, although only 2 of these described simultaneous deaths of ducks: 1 reported a duck flock death ratio of 80%, the other a ratio of 100%. Seven other households reported high levels of death among duck flocks (>60%), but the number of deaths among chickens did not suggest H5N1. Overall, raising ducks with chickens was not associated with deaths in chickens (p = 0.57).

Cloacal swabs were collected from 28 chickens and 14 ducks. Specimens from 2 sick chickens were positive for H5N1 by RT-PCR. These chickens belonged to a household located ≈50 m from the household of the confirmed human H5N1 case-patient. The owner of the sick chickens reported that the farmer with H5N1 virus infection spent daylight hours in his compound.

The space-time scan statistic detected a significant cluster of 25 (60%) households with an overall relative risk of 7.9 (log likelihood ratio 34.1, p = 0.001) (Figure 1). The cluster was confined to the period from February 25 through March 26, with a radius of 444 m, which contained both the household of the confirmed H5N1 case-patient and the house with the 2 H5N1-infected chickens.

### Seroepidemiologic Survey Findings

Among 93 households that were surveyed, 351 persons participated and 3 refused. An average of 4 people resided in each household, the median age was 23 years (range 1 month–81 years), and 150 (42.7%) of the sample were male; 207 (59%) were farmers of both crops and livestock. The rest of the participants were students (29.3%), had no stated occupation (18.8%), or were construction or factory workers (0.9%). Reflecting the rural setting and the common means of livelihood, ownership of animals, including poultry, was high (Table 1). The number of households with chickens decreased by 17.5% (p<0.001) after the outbreak, while the number of households that possessed ducks and pigs remained similar (–15.7% and 5.7%, p = 0.43 and 0.74, respectively).

**Table 1:** Comparison of animal exposures in households in which the likelihood of H5N1 outbreak among chickens was high (n = 96) and in households in which no chickens died (n = 166)*

Exposure	Likely H5N1 outbreak households, n (%)	No chicken death households, n (%)	OR	p value	95% CI
Handle live poultry	56 (58.3)	125 (75.3)	0.46	0.025	0.23–0.91
Feed poultry	63 (65.6)	130 (78.3)	0.53	0.093	0.25–1.11
Clean poultry cages and stalls	32 (33.3)	85 (51.2)	0.48	0.015	0.26–0.87
Collect sick poultry	53 (55.2)	86 (51.8)	1.15	0.623	0.66–1.98
Collect dead poultry	55 (57.3)	92 (55.4)	1.08	0.769	0.64–1.82
Pluck feathers from dead poultry	43 (44.8)	88 (53.0)	0.72	0.290	0.39–1.32
Handle poultry organs	51 (53.1)	107 (64.5)	0.62	0.100	0.36–1.09
Transport live poultry	3 (3.1)	7 (4.2)	0.73	0.647	0.19–2.77
Collect or transport feces	37 (38.5)	78 (47.0)	0.71	0.162	0.44–1.15
Raise hatchlings	1 (1.0)	0	NA		
Collect and sell eggs	36 (37.5)	69 (41.6)	0.84	0.585	0.46–1.55
Clean up poultry feathers	31 (32.3)	82 (49.4)	0.49	0.013	0.28–0.86
Clean up poultry feces	36 (37.5)	80 (48.2)	0.67	0.291	0.32–1.41
Slaughter chickens	30 (31.3)	68 (41.0)	0.40	0.017	0.18–0.87
Slaughter ducks	17 (17.7)	40 (24.1)	0.52	0.638	0.03–8.52
Attend cockfight	9 (9.4)	17 (10.2)	0.65	0.474	0.19–2.15
Purchase live poultry	11 (11.5)	6 (3.6)	3.45	0.028	1.14–10.44
Purchase killed poultry	2 (2.1)	0	NA		

*OR, odds ratio; CI, confidence interval; NA, not applicable.

Because many households owned poultry or pigs, a substantial proportion of the surveyed population had regular, high-intensity contact with these animals in the 12 months before the survey; this contact included collecting, processing, and eating sick birds or birds that had recently died when H5N1 viruses were thought to be circulating among flocks in the village. Despite this finding, none of the villagers interviewed reported having a febrile or respiratory illness during the same period, and none of the 351 participants had neutralizing antibodies suggestive of H5N1 virus infection on microneutralization assay.

We compared exposures of residents from households with a high probability of having had an outbreak of H5N1 in their chicken flock (n = 96) with occupants from households where no chickens died (n = 166) (Table 1). Bivariate analysis showed that households that purchased live poultry in the preceding year were almost 4× more likely to have had H5N1 in their flock than households that did not buy live chickens. In contrast, certain behavior by household members appeared to reduce the risk for H5N1 virus infection in their household flock by half (and the difference remained significant after controlling for poultry purchasing): cleaning cages or stalls, cleaning up poultry feathers, and handling live poultry (Table 1). Slaughtering chickens was not a significant risk factor after controlling for exposures that were significant on multivariate analysis (Table 2).

**Table 2:** Unconditional logistic regression models comparing households in which the likelihood of H5N1 outbreak among chickens was high and households in which no chickens died (n = 262)

Variable	Adjusted odds ratio	p value	Adjusted for variable nos.
1. Clean up cages/stalls	0.5	0.02	4, 5
2. Feed poultry	0.5	0.11	4, 5
3. Handle live poultry	0.4	0.03	4, 5
4. Purchase live poultry	4.5–4.9	<0.01	5 and (1 or 2 or 6)
5. Slaughter chickens	0.7–0.9	0.23–0.58	4 and (1 or 2 or 6)
6. Clean up poultry feathers	0.5	0.01	4, 5

## Discussion

The primary finding of our investigations is that transmission of H5N1 viruses from infected poultry to humans appears to have been low in a rural Cambodian population with confirmed and suspected H5N1 poultry outbreaks, and where a human H5N1 case occurred during 2005. This finding is consistent with other studies that have described low frequency of H5N1 neutralizing antibody among healthcare workers and household contacts since 2004 (*11**–**13*). Moreover, our findings suggest that asymptomatic and mild H5N1 virus infections had not occurred in the population we investigated. Although H5N1 virus was only isolated from birds in 1 household, evidence suggested an H5N1 outbreak among numerous chicken flocks in the village beginning ≈6 weeks before the human H5N1 case was confirmed. Given that direct contact with poultry and poultry products was common among people in this village, a high proportion of villagers were presumably exposed to H5N1 virus. Genetic analysis of H5N1 virus isolates from the infected farmer and 2 chickens confirmed that no reassortment with elements of human influenza A viral genome had occurred (*2*). We cannot say why illness developed in 1 person when family, neighbors, and many other villagers who reported similar poultry exposures did not have any evidence of H5N1 virus infection.

The seroprevalence of H5N1 antibody in the Cambodian population surveyed was substantially lower than was found in poultry workers in Hong Kong in 1997 with the same microneutralization assay (*13*). Although this assay is a highly specific and strain-dependent test and may not detect neutralizing antibody to antigenically distinct H5N1 virus strains, the 2004 Vietnam clade 1 H5N1 virus strain used in our investigation was antigenically identical and genetically similar to H5N1 viruses circulating among poultry in Cambodia in 2005 (*2*). Nonetheless, a small chance exists that previous H5N1 virus infection might have been missed if levels of H5N1 neutralizing antibodies had declined; for example, some human influenza virus infections do not invariably result in a detectable serum antibody response (*14*). However, the kinetics of the H5N1 antibody response in humans is similar to that of human influenza A virus (*15*). In addition, recent evidence shows that the H5N1 virus results in a systemic infection likely to produce a neutralizing antibody response (*16**,**17*). When these results are considered with our findings from a sample of >350 people, H5N1 virus infection was not likely to have occurred without any circulating immunoglobulin G, even 2 months after symptom onset. H5N1 virus transmission to humans may be rare because it only occurs in exposed persons with unique host susceptibilities and a predisposition to an abnormal inflammatory response that results in severe and fatal outcomes, rather than causing a broad spectrum of illness with mild disease and subclinical infections. Nevertheless, further research is needed to better understand the immune response to H5N1 virus infection in humans.

Our investigations also found that some animal-handling practices, such as handling poultry, cleaning poultry stalls and cages, and collecting poultry feathers appeared to reduce the chance that a flock would be infected by H5N1 virus. This finding is in contrast to findings that handling dead or sick poultry is a risk factor for (individual) human H5N1 illness (*18**,**19*). We speculate that some practices that encourage backyard birds to stay close to the house, such as handling poultry, may be protective by reducing the distance healthy fowl need to roam to forage for food, thereby reducing interactions with wild and other domestic birds and contact with contaminated environments. Cleaning poultry stalls and cages and collecting poultry feathers may indicate a better level of general hygiene practices and may also decrease the risk by removing potentially infectious materials. These findings may highlight the value of educating farmers about hygienic animal-handling practices.

Other behavior appeared to modify the risk for H5N1 in domestic fowl. Purchasing live poultry increased risk. The introduction of new birds that may be harboring disease is an obvious threat to a flock. Anecdotal evidence suggests that farmers in Kampot Province had responded to the culling without compensation control measures by attempting to sell birds at the first sign of sickness during outbreaks in 2005 (World Health Organization, unpub. data). Additionally, poultry trade with Vietnamese farmers was common and persisted despite the introduction of laws prohibiting such cross-border trafficking. Southern Vietnam has, like Cambodia, experienced mass H5N1 outbreaks among domestic fowl in the last few years (World Health Organization, unpub. data), and the village examined in this survey was 20 km from the border.

Our results need to be interpreted in the context of several limitations. The interview process involved a recall period of 12 months and did not document more temporally relevant exposures immediately before or during the outbreak. The long recall period may increase the probability of exposure to potential risk factors, making households with and without suspected H5N1 virus infection in flocks more similar. In addition, the temporal association between behavioral risk factors and H5N1 virus infection in poultry was difficult to establish. On the basis of our counterintuitive findings, further risk factor studies should address this potential bias. The classification of households as likely having H5N1 in their flocks was based on a case definition suggested by Food and Agriculture Organization veterinarians. Without confirmation of H5N1 virus infection, we do not know the sensitivity and specificity of this definition and cannot quantify the degree of misclassification, if any. The study did not collect information about the origins of each flock, how long birds had belonged to each household, or internal movements of individual birds and flocks within the village. This limitation hindered mapping the likely circulation of H5N1 virus among poultry in the village, and other factors related to poultry transmission may have been missed. Although we did not record and map households we did not visit, this bias is likely to have been nondifferential because the proportions of nonvisited households were similar for all 4 investigation teams that surveyed in 4 different directions. Finally, specimen collection had limitations. Higher concentrations of virus exist in the trachea of infected birds rather than in the cloacae (*20*), but tracheal sampling was not performed in this survey because it was not acceptable to local farmers.

This study provides evidence of the low transmissibility of the H5N1 virus from infected poultry to humans, even in circumstances in which human-poultry interactions are regular and intense. In this instance, human H5N1 virus infection manifested as a single case of severe illness without any evidence that the virus could cause either mild disease or asymptomatic infection. However, our findings are limited to the investigation period of 2005. As H5N1 viruses continue to circulate and evolve among poultry, poultry-to-human transmission of H5N1 viruses could increase. Extensive investigations should be routinely conducted for all H5N1 outbreaks among humans and animals to monitor the nature and extent of bird-to-human or human-to-human transmission of H5N1 viruses. Additional seroepidemiologic investigations should be conducted to assess the ongoing risk for bird-to-human transmission of H5N1 among rural and other human populations.
